# Risk factors of multidrug-resistant organisms neonatal sepsis in Surabaya tertiary referral hospital: a single-center study

**DOI:** 10.1186/s12887-024-04639-9

**Published:** 2024-02-29

**Authors:** Stefani Miranda, Aminuddin Harahap, Dominicus Husada, Fara Nayo Faramarisa

**Affiliations:** 1 Department of Child Health, Faculty of Medicine, Hang Tuah University/dr. Ramelan Navy Central Hospital, Jalan Gadung No. 1, Surabaya, East Java, 60244 Indonesia; 2 Department of Child Health, dr. Ramelan Navy Central Hospital, Jalan Gadung No.1, Surabaya, East Java, 60244 Indonesia; 3 Department of Clinical Microbiology, dr. Ramelan Navy Central Hospital, Jalan Gadung No.1, Surabaya, East Java, 60244 Indonesia; 4 Department of Child Health, Faculty of Medicine, Universitas Airlangga/Dr. Soetomo Academic General Hospital, Jalan Prof. Dr. Moestopo 6-8, Surabaya, East Java, 60286 Indonesia

**Keywords:** MDRO, Neonatal sepsis, Newborn, Risk factors

## Abstract

**Background:**

Bacterial organisms causing neonatal sepsis have developed increased resistance to commonly used antibiotics. Antimicrobial resistance is a major global health problem. The spread of Multidrug-Resistant Organisms (MDROs) is associated with higher morbidity and mortality rates. This study aimed to determine the risk factors for developing MDRO neonatal sepsis in the Neonatal Intensive Care Unit (NICU), dr. Ramelan Navy Central Hospital, in 2020–2022.

**Methods:**

A cross-sectional study was performed on 113 eligible neonates. Patients whose blood cultures were positive for bacterial growth and diagnosed with sepsis were selected as the study sample. Univariate and multivariate analysis with multiple logistic regression were performed to find the associated risk factors for developing multidrug-resistant organism neonatal sepsis. A *p*-value of < 0.05 was considered significant.

**Results:**

Multidrug-resistant organisms were the predominant aetiology of neonatal sepsis (91/113, 80.5%). The significant risk factors for developing MDRO neonatal sepsis were lower birth weight (OR: 1.607, 95% CI: 1.003 − 2.576, *p*-value: 0.049), history of premature rupture of the membrane (ProM) ≥ 18 (OR: 3.333, 95% CI: 2.047 − 5.428, *p*-value < 0.001), meconium-stained amniotic fluid (OR: 2.37, 95% CI: 1.512 − 3.717, *p*-value < 0.001), longer hospital stays (OR: 5.067, 95% CI: 2.912 − 8.815, *p*-value < 0.001), lower Apgar scores (OR: 2.25, 95% CI: 1.442 − 3.512, *p*-value < 0.001), and the use of respiratory support devices, such as invasive ventilation (OR: 2.687, 95% CI: 1.514 − 4.771, *p*-value < 0.001), and non-invasive ventilation (OR: 2, 95% CI: 1.097 − 3.645, *p*-value: 0.024).

**Conclusions:**

Our study determined various risk factors for multidrug-resistance organism neonatal sepsis and underscored the need to improve infection control practices to reduce the existing burden of drug-resistant sepsis. Low-birth-weight, a maternal history of premature rupture of the membrane lasting more than 18 hours, meconium-stained amniotic fluid, longer hospital stays, a low Apgar score, and the use of ventilators were the risk factors for developing drug-resistant neonatal sepsis.

**Supplementary Information:**

The online version contains supplementary material available at 10.1186/s12887-024-04639-9.

## Background

Neonatal sepsis is a clinical syndrome characterized by systemic signs and symptoms of infection and is accompanied by bacteremia within the first month of life [1]. Neonatal sepsis necessitates intense treatment in the NICU due to respiratory distress and hemodynamic instability [2]. Infants in the NICU are at high risk of multidrug-resistant organisms’ infection due to their small gestational age, low body weight, poor resistance, critical condition, high incidence of invasive procedures, prolonged hospital stays (≥ 5 days), long-term antimicrobial therapy, post-surgical operations, and decreased compliance with infection control measures [3–6]. Sepsis caused by MDRO was associated with a higher mortality rate in neonate population [7].

Neonatal sepsis is the third-most common cause of neonatal death, according to the World Health Organization (WHO) [1]. A meta-analysis from 14 countries carried out by Fleischmann C. et al. (2021) found an incidence of 2824 neonatal sepsis cases per 100.000 live births. The mortality rate was 17.6% [8]. Bacterial organisms that cause neonatal sepsis have become more resistant to commonly used antibiotics [9]. Multidrug-resistant organisms spread is associated with increased costs as well as higher morbidity and mortality rates. Antimicrobial resistance is a major global health issue. The WHO has defined the fight against MDRO spread as a “critical” priority [10].

When pathogens enter the body of a newborn, they can cause an infection. It can lead to sepsis if it is not prevented because babies lack an immune system capable of fighting infection [11]. Bacteria develop resistance to antimicrobial drugs through well-defined mechanisms such as enzyme inactivation, changed target sites, efflux, and altered membrane permeability. Antimicrobial drug resistance in hospitals was driven by hospital hygiene failures, selective pressures produced by antibiotic overuse, and mobile genetic elements carrying resistance genes that are transmitted across particular bacterium species [12, 13]. Antimicrobial stewardship programs implementation in conjunction with proper empirical antibiotic regimens will reduce selective pressure on resistant strains and their subsequent deleterious effects in neonates [14]. NICUs should strive to develop effective antimicrobial stewardship through coordinated interventions with other teams such as microbiology, paediatric infectious diseases, and pharmacists [14–16].

Multiple pathogens were identified by culture. According to the Global Antibiotic Research & Development Partnership (GARDP) neonatal study report (2016), *Klebsiella pneumoniae* was the most common pathogen isolated and is usually associated with hospital-acquired infections, which are increasingly resistant to existing antibiotic treatments [17]. It is important to understand the risk factors that lead to drug-resistant neonatal septicemia in order to develop management strategies. As a result, the purpose of this study was to investigate the risk factors for developing multidrug-resistant organisms septicemia in neonates in our NICU. It is hoped that our study will provide a comprehensive picture of the risk factors for developing MDROs septicemia in neonates, allowing its incidence to be reduced by avoiding modifiable risk factors.

## Methods

### Study overview

This is a cross-sectional study that uses secondary data from dr. Ramelan Navy Central Hospital’s NICU and clinical microbiology department in Surabaya, Indonesia. This hospital is the highest tertiary naval hospital in East Java. The NICU at this hospital has a total capacity of 18 beds and an annual capacity of 810 neonates. This hospital has 12 paediatricians on staff, including one neonatologist and one paediatric cardiologist. Infants who presented with clinical signs and symptoms of sepsis had blood cultures taken. According to the Canadian Paediatric Society’s guideline [18], clinical manifestations of neonatal sepsis may be subtle and include an abnormal heart rate, poor peripheral perfusion, temperature instabilities, and signs of respiratory distress.

### Population and samples

#### Inclusion criteria

This study included sepsis neonates treated in dr. Ramelan Navy Central Hospital’s NICU, whose blood drawn for culture.

#### Exclusion criteria

This study excluded neonates whose blood culture results were negative and showed fungal growth.

### Definitions

Early-onset sepsis: blood culture–proven infection occurring in the newborn at < 7 days of age [19].

Late-onset sepsis: blood culture–proven infection occurring in the newborn after 7 days of age caused by a postnatal acquisition (nosocomial or community sources) [19].

MDRO: organisms with acquired non-susceptibility to at least one agent in three or more antimicrobial categories [20].

Neonate: a newborn or an infant within its first 28 days.

Neonatal sepsis: a clinical syndrome of systemic illness accompanied by bacteremia occurring in the first month of life [19].

### Sample size determination

The sample size for this study was calculated manually using a single proportion formula. The proportion of neonatal sepsis was derived from a study conducted in Eastern Saudi Arabia, which had a proportion of 5.16% [5]. With a margin of error of 5% and a confidence interval of 95%, this study’s final sample size was 76 neonates.

### Sampling techniques

Total sampling was used as the sampling technique, and the samples were taken sequentially. We included all neonates admitted to our NICU between January 1, 2020 and December 31, 2022 who met the inclusion and exclusion criteria, as we could meet the sample size previously estimated for this study.

### Data collection and management

Data was collected from the clinical microbiology department’s data entry and patients’ electronic medical records using a pre-designed data extraction form. The tools contained information on the characteristics of the patients, such as gender, onset of sepsis, birth weight, gestational age, Lubchenco curve, mode of delivery, history of premature rupture of the membrane for more than 18 hours, type of amniotic fluid, history of antibiotic use during delivery, congenital malformations, admission type, length of hospital stay, Apgar score at 5 minutes, history of using respiratory support devices, history of being placed on a vascular access, and the outcome.

The clinical microbiology department’s data entry was utilized to obtain a list of culture examination requests for neonatal patients. From these lists, patients with positive blood cultures for bacterial growth were selected. The medical record numbers of these patients were recorded, and then the diagnosis of sepsis was manually searched for in the patients’ medical records. The attending paediatrician was responsible for making the sepsis diagnosis. Neonatal sepsis patients with an International Classification of Diseases (ICD)-10 code of P36.0 to P36.8 were included in this study. The medical records of chosen patients were reviewed to gather information about the patients’ characteristics.

### Data analysis

Descriptive analysis was carried out by determining the frequency distributions of the patient’s characteristics based on blood culture results (MDRO vs. non-MDRO). A univariate analysis was used to compare the two groups using the chi-square test, with blood culture results as the independent variable. After identifying significant risk factors, a multivariate analysis was performed using multiple logistic regression with the “enter” method to determine associated risk factors. The study reported the odds ratio (OR) and 95% confidence intervals (CIs). A significance level of *p* < 0.05 was used. All analyses were performed using SPSS Version 29 (SPSS Inc., Chicago, IL).

## Results

### Searching for medical records

**Fig. 1:**
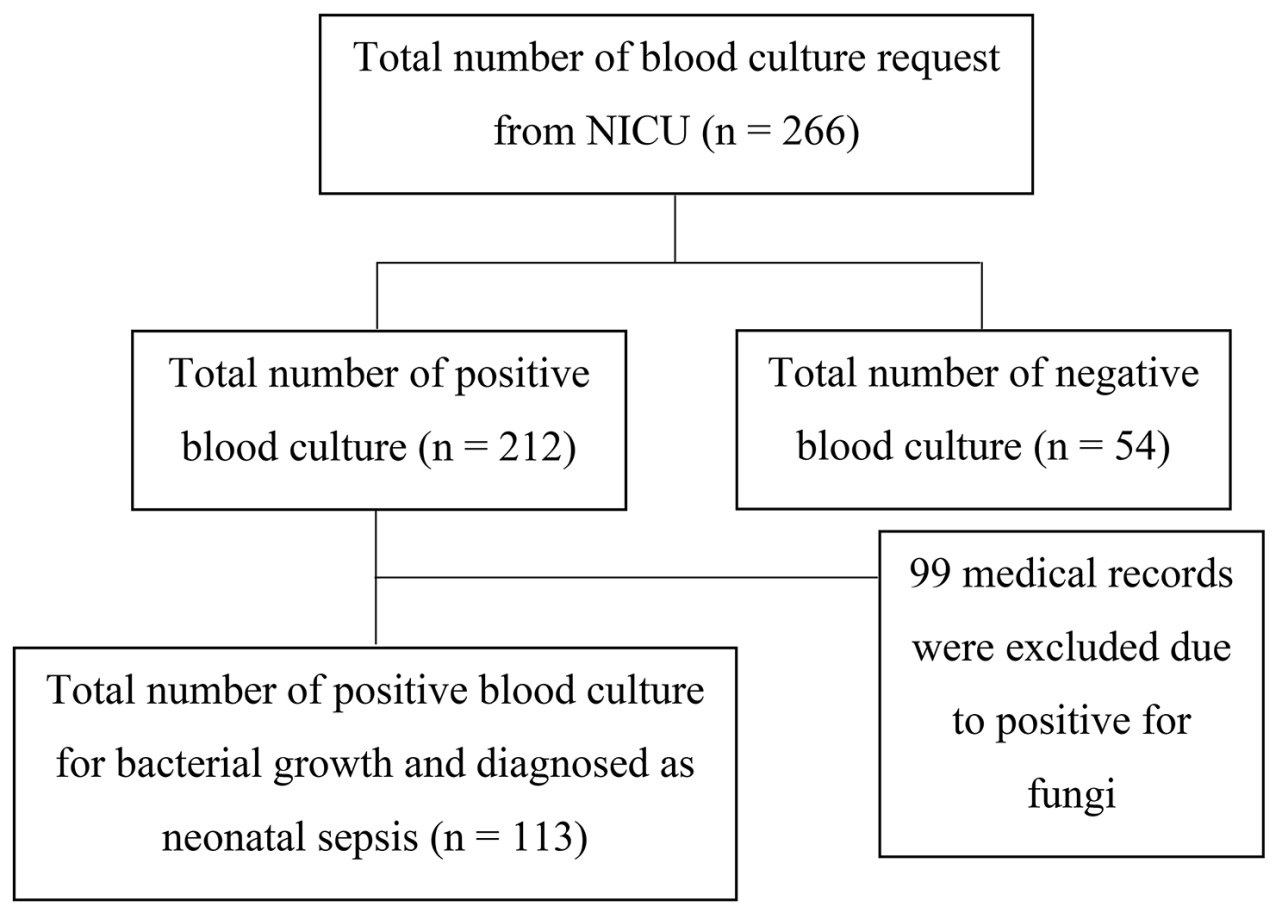
The flow of medical records search

This study explored 266 electronic medical records from dr. Ramelan Navy Central Hospital’s NICU in Indonesia. The study’s final subjects consisted of 113 neonates who had sepsis diagnosed by each attending physician and confirmed by a blood culture examination. From the 212 total positive blood cultures, 99 neonates were excluded because they had isolates positive for fungi growth. The process of looking for medical records is depicted in Fig. 1.

### Demographic features

Over a span of three years, the NICU at dr. Ramelan Navy Central Hospital requested 266 blood culture examinations, accounting for nearly half (113/266, 42.5%) of all examination requests. This study revealed that multidrug-resistant organisms were prevalent (91/113, 80.5%). Further details about the MDROs can be found in the Supplementary Material (Table S1). The majority of the patients at this hospital were male (59/113, 52.2%), weighed between 1500 and 2499 g (50/113, 44.2%), and were delivered via caesarean Sect. (71/113, 62.8%). Annually, 430 male neonates were admitted to the NICU at this hospital.

### Risk factors associated with the occurrence of MDRO neonatal sepsis

We performed a univariate analysis of the risk factors observed in our study, as shown in Table 1. All of the significant variables were subjected to a multiple logistic regression test to determine which of the risk factors increased the odds of multidrug-resistant organism neonatal sepsis (Table 2). The risk of MDRO neonatal sepsis is significantly increased by several factors, including low-birth-weight, a maternal history of premature rupture of the membrane lasting more than 18 hours, meconium-stained amniotic fluid, longer hospital stays, a low Apgar score, and the use of ventilators. In particular, lower birth weight (OR: 1.607, 95% CI: 1.003 − 2.576, *p*-value: 0.049) and premature rupture of the membrane lasting more than 18 hours (OR: 3.333, 95% CI: 2.047 − 5.428, *p*-value < 0.001) are associated with a higher risk of MDRO neonatal sepsis. Meconium-stained amniotic fluid (OR: 2.37, 95% CI: 1.512 − 3.717, *p*-value < 0.001) also poses a greater risk compared to clear amniotic fluid. Additionally, longer hospital stays (OR: 5.067, 95% CI: 2.912 − 8.815, *p*-value < 0.001) and lower Apgar scores (OR: 2.25, 95% CI: 1.442 − 3.512, *p*-value < 0.001) are linked to an increased risk. Furthermore, the use of more invasive respiratory support devices, such as invasive ventilation (OR: 2.687, 95% CI: 1.514 − 4.771, *p*-value < 0.001), further raises the risk of MDRO neonatal sepsis, compared to non-invasive ventilation (OR: 2, 95% CI: 1.097 − 3.645, *p*-value: 0.024).

**Table 1:** Characteristics of the study population

Variables	Category	MDRO	Non-MDRO	*p* value	
		**Frequency (%)**	**Frequency (%)**		
Gender	Female	47 (51.6)	7 (31.8)	0.095	
	Male	44 (48.4)	15 (68.2)		
Onset of sepsis	EOS	55 (60.4)	9 (40.9)	0.097	
	LOS	36 (39.6)	13 (59.1)		
Birth weight (grams)	≥ 2500	28 (30.8)	7 (31.9)	0.045*	
	1500–2499	45 (49.5)	5 (22.7)		
	1000–1499	11 (12.1)	5 (22.7)		
	< 1000	7 (7.6)	5 (22.7)		
Gestational age	Term	37 (40.7)	15 (68.2)	0.02*	
	Preterm	54 (59.3)	7 (31.8)		
Lubchenco curve	AGA	64 (70.3)	9 (40.9)	0.011*	
	SGA	20 (22)	7 (31.8)		
	LGA	7 (7.7)	6 (27.3)		
Mode of delivery	Normal labour	34 (37.4)	8 (36.4)	0.931	
	Caesarean section	57 (62.6)	14 (63.6)		
ProM more than 18 h	No	21 (23.1)	12 (54.5)	0.004*	
	Yes	70 (76.9)	10 (45.5)		
Amniotic fluid	Clear	27 (29.7)	12 (54.5)	0.028*	
	Meconeal	64 (70.3)	10 (45.5)		
Antibiotic use during delivery	No	32 (35.2)	7 (31.8)	0.767	
	Yes	59 (64.8)	15 (68.2)		
Congenital malformation	No	70 (76.9)	12 (54.5)	0.035*	
	Yes	21 (23.1)	10 (45.5)		
Admission type	Inborn	62 (68.1)	9 (40.9)	0.018*	
	Outborn	29 (31.9)	13 (59.1)		
Length of hospital stay (days)	≤ 7	15 (16.5)	16 (72.7)	< 0.001*	
	> 7	76 (83.5)	6 (27.3)		
Apgar score at 5 min	≥ 7	28 (30.8)	13 (59.1)	0.013*	
	< 7	63 (69.2)	9 (40.9)		
Respiratory support device	None	16 (17.6)	9 (40.9)	0.049*	
	NIV	32 (35.2)	7 (31.8)		
	IV	43 (47.2)	6 (27.3)		
Vascular access	Peripheral vascular access	60 (65.9)	15 (68.2)	0.841	
	Central vascular access	31 (34.1)	7 (31.8)		
Outcome	Live	42 (46.2)	16 (72.7)	0.025*	
	Death	49 (53.8)	6 (27.3)		

Notes: *representing the significant *p*-value

*EOS* Early-onset sepsis, *LOS* Late-onset sepsis, *SGA* Small for gestational age, *AGA* Appropriate for gestational age, *LGA* Large for gestational age, *ProM* Premature rupture of the membrane, *NIV* Non-invasive ventilation, *IV* Invasive ventilation

**Table 2:** Multivariate analysis for determining odds ratio of potential risk factors for MDRO neonatal sepsis

Variables	Category	OR	95% CI	*p* value
Birth weight (grams)	≥ 2500	1	Reference	
	1500–2499	1.607	1.003 − 2.576	0.049*
	1000–1499	0.393	0.196 − 0.789	0.009*
	< 1000	0.25	0.109 − 0.572	0.001*
Gestational age	Term	1	Reference	
	Preterm	1.459	0.961 − 2.217	0.076
Lubchenco curve	AGA	1	Reference	
	SGA	0.312	0.189 − 0.516	< 0.001*
	LGA	0.109	0.05 − 0.239	< 0.001*
ProM more than 18 h	No	1	Reference	
	Yes	3.333	2.047 − 5.428	< 0.001*
Amniotic fluid	Clear	1	Reference	
	Meconeal	2.37	1.512 − 3.717	< 0.001*
Congenital malformation	No	1	Reference	
	Yes	0.3	0.184 − 0.489	< 0.001*
Admission type	Inborn	1	Reference	
	Outborn	0.468	0.301 − 0.727	< 0.001*
Length of hospital stay (days)	≤ 7	1	Reference	
	> 7	5.067	2.912 − 8.815	< 0.001*
Apgar score at 5 min	≥ 7	1	Reference	
	< 7	2.25	1.442 − 3.512	< 0.001*
Respiratory support device	None	1	Reference	
	NIV	2	1.097 − 3.645	0.024*
	IV	2.687	1.514 − 4.771	< 0.001*

Notes: *representing the significant *p*-value

*EOS* Early-onset sepsis, *LOS* Late-onset sepsis, *SGA* Small for gestational age, *AGA* Appropriate for gestational age, *LGA* Large for gestational age, *ProM* Premature rupture of the membrane, *NIV* Non-invasive ventilation, *IV* Invasive ventilation

## Discussion

Septicemia is still one of the leading causes of neonatal mortality. Understanding the pathogens responsible for neonatal septicemia is essential for developing management strategies, particularly empirical antibiotic regimens [21]. Early initiation of appropriate antibiotic therapy is critical for improving the outcome and success of neonatal sepsis treatment [9]. Broad-spectrum antibiotic overuse and misuse have been linked to the emergence of antibiotic-resistant pathogens [22]. The clinical setting is becoming increasingly associated with the development of antimicrobial resistance due to the high level of antibiotic use [23]. Multidrug-resistance organism are recognized as hospital-acquired pathogens, and they impose a significant financial burden on healthcare systems [5].

In our study, low birth weight proved to be a significant risk factor for the development of multidrug-resistant organism neonatal sepsis. The majority of the neonates had a low birth weight (50/113, 44.2%). Among the low-birth-weight neonates, the rate of multidrug resistance was notably high (45/50, 90%). A study from Ethiopia indicated that low-birth-weight neonates had a higher likelihood of developing drug-resistant neonatal sepsis compared to those with a normal birth weight [24]. This finding was also supported by other studies [25–27]. However, a study conducted by Licona G. et al. (2023) concluded that birth weight was not a risk factor for developing drug-resistant neonatal sepsis [28].

A maternal history of premature rupture of the membrane lasting over 18 hours was identified as a significant risk factor for the development of multidrug-resistance organism neonatal sepsis. The majority of the neonates had a maternal history of ProM lasting over 18 hours (80/113, 70.8%), and the rate of multidrug resistance among this group was considered high (70/80, 87.5%). Early and prolonged rupture of the membranes increases the risk of ascending infection from the birth canal into the amniotic sac and foetal membrane, potentially causing asphyxia, which frequently causes sepsis [29]. In a study by Awad HA. et al. (2016), a maternal history of ProM was significantly associated with the development of MDRO neonatal sepsis (*p*-value < 0.001) [9]. Several studies reported that a maternal history of ProM was not a significant risk factor for developing MDRO neonatal sepsis [27, 30, 31]. Neonates born with meconium-stained amniotic fluid were at risk for developing multidrug-resistance organism neonatal sepsis. The majority of the neonates in our research were born with meconium-stained amniotic fluid (74/113, 65.5%), and the rate of multidrug-resistance was high among this group (64/74, 86.5%). Consistent with our findings, a study in Surabaya also identified meconium-stained amniotic fluid as a risk factor for developing neonatal sepsis [32]. Conversely, a case-control study of 248 neonates in southwest Ethiopia found that meconium-stained amniotic fluid was not a risk factor for developing neonatal sepsis [12]. Manandhar S. et al. (2021) reported the same finding in Nepal [33].

Our study found that longer hospital stays increased the risk of multidrug-resistance organism neonatal sepsis. The majority of the neonates stayed in our hospital for longer than 7 days (82/113, 72.6%). The rate of multidrug-resistance was high in that group (76/82, 92.7%). Prolonged hospital stays (≥ 5 days) were identified as one of the potential risk factors for developing hospital-acquired MDRO neonatal sepsis in a single-center study in Saudi Arabia [6]. Another study reached the same conclusion [22, 33–35]. Exposure to healthcare is a risk factor for acquiring colonization or infection with antibiotic-resistant bacteria due to cross-transmission or selection pressure on the microbiome during antibiotic treatment. Conversely, an infection with a resistant strain may lengthen the hospital stay because it is more difficult to treat [36]. The Tuzla study revealed that neonates with MDR sepsis required more intense care (20.7 ± 10.8 vs. 12.4 ± 6.93 days) than the non-MDR sepsis group (*p*-value < 0.001) [30]. Bandyopadhyay T. et al. (2018) proved that duration of hospital stay was not a significant risk factor for developing MDRO neonatal sepsis, with a median hospital stay of 22 days [37].

Lower Apgar scores were associated with an increased risk of multidrug-resistant organism neonatal sepsis. The majority of the neonates in the study were born with an Apgar score of less than 7 at 5 minutes (72/113, 63.7%), and multidrug resistance was prevalent in neonates from this group (63/72, 87.5%). This finding aligns with a study conducted in Ghana (*p*-value < 0.001) [38]. However, G/eyesus T. et al. (2017) reported the opposite, concluding that a low Apgar score was not a risk factor for developing neonatal sepsis (*p*-value 0.023) [39]. Additionally, the use of more invasive respiratory support devices was found to increase the risk of multidrug-resistant organism neonatal sepsis. A significant proportion of the neonates in the study relied on invasive ventilation as their respiratory support (49/113, 43.4%). Multidrug resistance was prevalent among neonates using invasive ventilation (43/49, 87.8%). Neonates on invasive ventilation faced a 2.687-fold greater risk of developing MDRO neonatal sepsis compared to those receiving non-invasive ventilation. Neonates with non-invasive ventilation were at a 2-fold risk of developing MDRO neonatal sepsis. A study conducted in eastern Saudi Arabia revealed that the use of a mechanical ventilator for ≥ 72 h significantly increased the risk of MDR neonatal sepsis (*p*-value 0.049) [5]. This finding was supported by other studies [26, 40].This study’s strength was that we explored numerous variables as risk factors for developing multidrug-resistance organism neonatal sepsis, especially the Lubchenco curve as one of the variables, which is still seldom observed in previous studies. As a result of this study, it is envisaged that the incidence of MDRO neonatal sepsis might be reduced by avoiding modifiable risk factors. Our hospital has its own obstetric ward, and nearly all neonates were born there. This makes the related data obtained for this study more precise and representative, as incomplete data from referral patients due to missed communications could be limited. Although this was a thorough study, it has some limitations. First, because this was a single-center study, the findings may be less generalizable to other institutes. Second, misclassification or recollection bias could arise because this was a retrospective medical record-based study.

## Conclusions

Our study determined the burden, demographics, and risk factors for multidrug-resistance organism neonatal sepsis. The majority of the isolates in this study grew for MDRO. Low-birth-weight, a maternal history of premature rupture of the membrane lasting more than 18 hours, meconium-stained amniotic fluid, longer hospital stays, a low Apgar score, and the use of ventilators were found to be significant independent risk factors for developing drug-resistant neonatal sepsis.

Continuous surveillance for antibiotic susceptibility is required to maintain optimal empirical antibiotic therapy. Antibiotic susceptibility testing should be used to assess whether antibiotics should be kept, changed, or terminated. Improvement of infection control practices, avoidance of irrational use of antibiotics, and revision of the protocols for treatment are mandatory to prevent further resistance. Auditing and continuous quality improvement programs are essential. A more representative multi-center study is expected and should be carried out in the future.

### Electronic supplementary material

Below is the link to the electronic supplementary material.

Supplementary Material 1

Supplementary Material 2

## Data Availability

The datasets supporting the conclusion of this article are included within the article and its additional files.

## Abbreviations

AGA: Appropriate for Gestational Age
AST: Antimicrobial Susceptibility Testing
CI: Confidence Interval
EOS: Early-Onset Sepsis
ICD: International Classification of Diseases
IV: Invasive Ventilation
LGA: Large for Gestational Age
LOS: Late-Onset Sepsis
MDR: Multi Drug-Resistance
MDROs: Multi Drug-Resistant Organisms
ml: millilitre
NICU: Neonatal Intensive Care Unit
NIV: Non-Invasive Ventilation
OR: Odds Ratio
ProM: Premature rupture of the Membrane
SGA: Small for Gestational Age
SPSS: Statistical Package for the Social Sciences
WHO: World Health Organization
