# Supplementary material for: Risk factors of multidrug-resistant organisms neonatal sepsis in Surabaya tertiary referral hospital: a single-center study
Source: BMC Pediatr. 2024 Feb 29;24:153. doi: 10.1186/s12887-024-04639-9 (PMC10902940; doi:10.1186/s12887-024-04639-9)
Supplement: Supplementary file 2 — Supplementary Material 2 [file 12887_2024_4639_MOESM2_ESM.docx]

# Supplementary information

File name: Table S1

File format: .xlsx

Title of the data: Table S1 The percentage of antimicrobial resistance in this study.

Description of the data: Table S1 showed the details of multidrug-resistant organisms found in this study.
